# Supplementary material for: Amorphous/crystalline interwoven multipods with high Co/Ni activity for wide-temperature-range sodium-sulfur batteries
Source: Nat Commun. 2026 Mar 10;17:2333. doi: 10.1038/s41467-026-69749-7 (PMC12979862; doi:10.1038/s41467-026-69749-7)
Supplement: Supplementary file 1 — Supplementary Information [file 41467_2026_69749_MOESM1_ESM.pdf]

Supplementary Information for

Amorphous/crystalline interwoven multipods with high Co/Ni activity for wide-temperature-range sodium-sulfur batteries

Tingjiao Xiao[1,2,†], Zhen Fang[3,4,5,†], Nian Ran[6,†], Ronghui Liu[1,2], Yuxuan Gao[7], Jianbo Wu[3,4,5], Jianjun Liu[6], Hua Wang[2], Wen-Feng Lin[8], and Wei Zhou[1,2,8]*

[1]Hangzhou International Innovation Institute, Beihang University, Hangzhou 311115, China

[2]School of Chemistry, Beihang University, Beijing 100191, China

[3]Center of Hydrogen Science & State Key Laboratory of Metal Matrix Composites, School of Materials Science and Engineering, Shanghai Jiao Tong University, Shanghai 200240, China

[4]Future Material Innovation Center, Zhangjiang Institute for Advanced Study, Shanghai Jiao Tong University, Shanghai 200240, China

[5]Materials Genome Initiative Center, Shanghai Jiao Tong University, Shanghai 200240, China

[6]State Key Laboratory of High Performance Ceramics, Shanghai Institute of Ceramics, Chinese Academy of Sciences, Shanghai 200050, China

[7]School of Materials Science and Engineering, Beihang University, Beijing 100191, China

[8]Department of Chemical Engineering, Loughborough University, Loughborough LE11 3TU, U.K.

[†]These authors contributed equally to this work

*Corresponding author. Email: W. Z. (zhouwei@buaa.edu.cn)

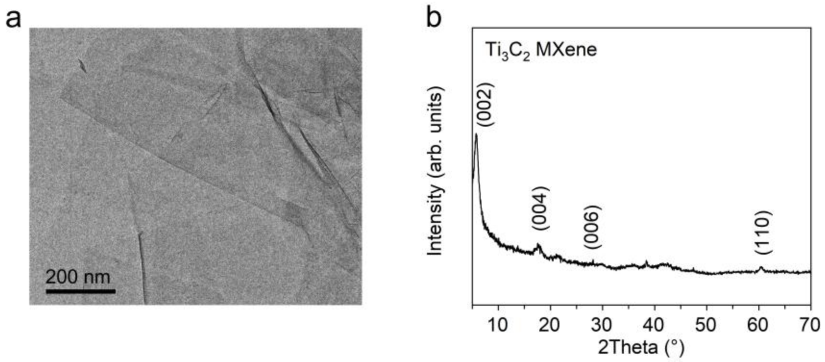

Supplementary Fig. 1 | Characterizations of $Ti_3C_2T_x$. a TEM image and **b** XRD pattern of $Ti_3C_2T_x$. Supplementary Fig. 1a reveals its characteristic layered structure. Supplementary Fig. 1b matches the $Ti_3C_2T_x$ MXene phase with diffraction peaks of (002), (004), (006) and (110) planes at 5.7°, 17.6°, 20.4°, and 28.1° respectively, confirming its crystalline structure [1,2].

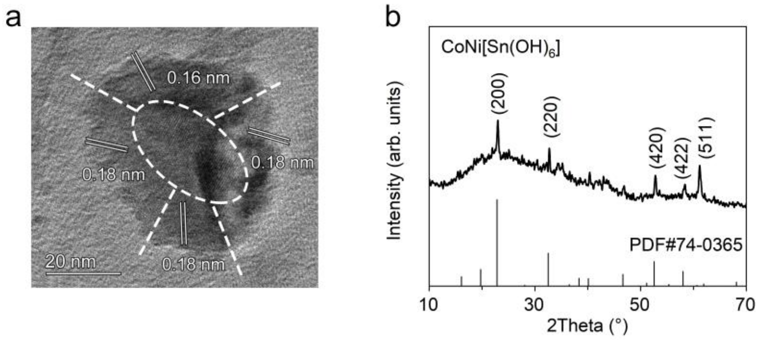

Supplementary Fig. 2 | Characterizations of precursor $Co_{0.5}Ni_{0.5}Sn(OH)_6$ grown on $Ti_3C_2T_x$. **a** SEM image and **b** XRD pattern of $Co_{0.5}Ni_{0.5}Sn(OH)_6$.

HRTEM image of the $Co_{0.5}Ni_{0.5}Sn(OH)_6$ precursor with spacing of 0.16 and 0.18 nm, corresponding to the planes of (422) and (420) separately. The particle is composed of five singe-crystal grains with different crystal growth orientation, which might serve as crystal seed for further formation of multipod-like structure [3]. XRD pattern matches the cubic $Co_{0.5}Ni_{0.5}Sn(OH)_6$ phase (JCPDS No. 74-0365) very well with diffraction peaks of (200), (220), (420), (422), and (511) planes at 22.8°, 32.5°, 52.6°, and 58.0° respectively.

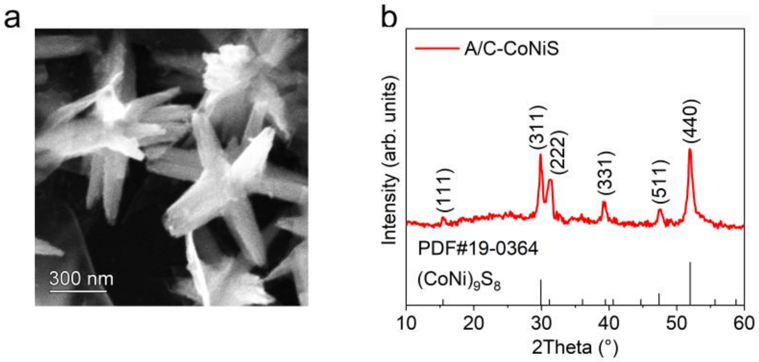

Supplementary Fig. 3 | Characterizations of A/C-CoNiS a SEM image and **b** XRD pattern of C-CoNiS.

Supplementary Fig. 3a exhibits a distinctive multipod morphology with radially extended branches. In Supplementary Fig. 3b, the diffraction peaks match well with cubic Co_9S_8 with planes of (111), (311), (222), (331), (511), and (440) (JCPDS No. 19-0364). The synthesized $(CoNi)_9S_8$ by adding nickel elements has the same atomic structure as that of Co_9S_8. The low degree of crystallinity indirectly confirms the amorphous-crystalline interwoven structure.

Supplementary Table 1.

Molar ratios of the elements in A/C-CoNiS determined by ICP-AES.

Elements	Co	Ni	Sn	S	Ti
Weight ratio	30.1%	26.4 %	5.5 %	27 %	11 %
Molar ratio	24.5 %	21.7 %	2.2 %	40.5 %	11.1 %

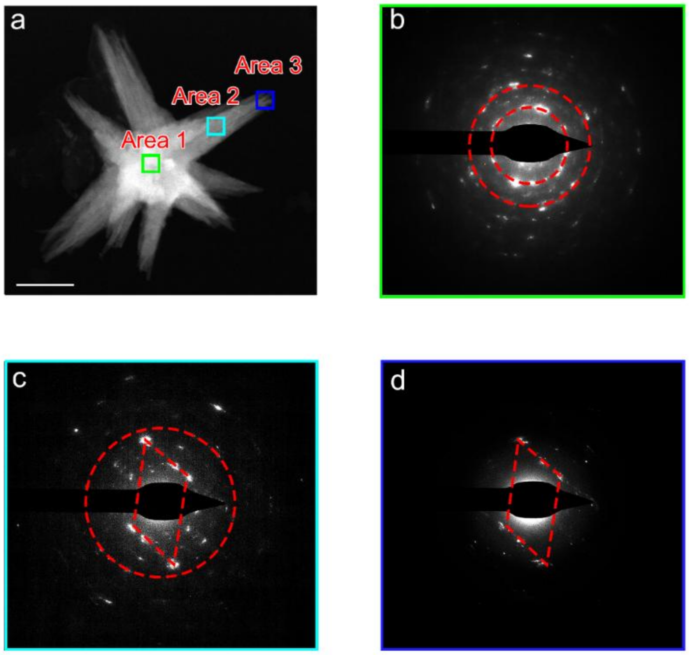

Supplementary Fig. 4 | Structural characterization of A/C-CoNiS. a HAADF-STEM image of A/C-CoNiS, **b–d** SAED patterns correspond to Areas 1–3 respectively.

SAED patterns (Supplementary Figs. 4b–d) indicate the multipod has a polycrystalline core and single-crystalline tips. We can deduce the branches might be assembled by single-crytal quasi-1D structures.

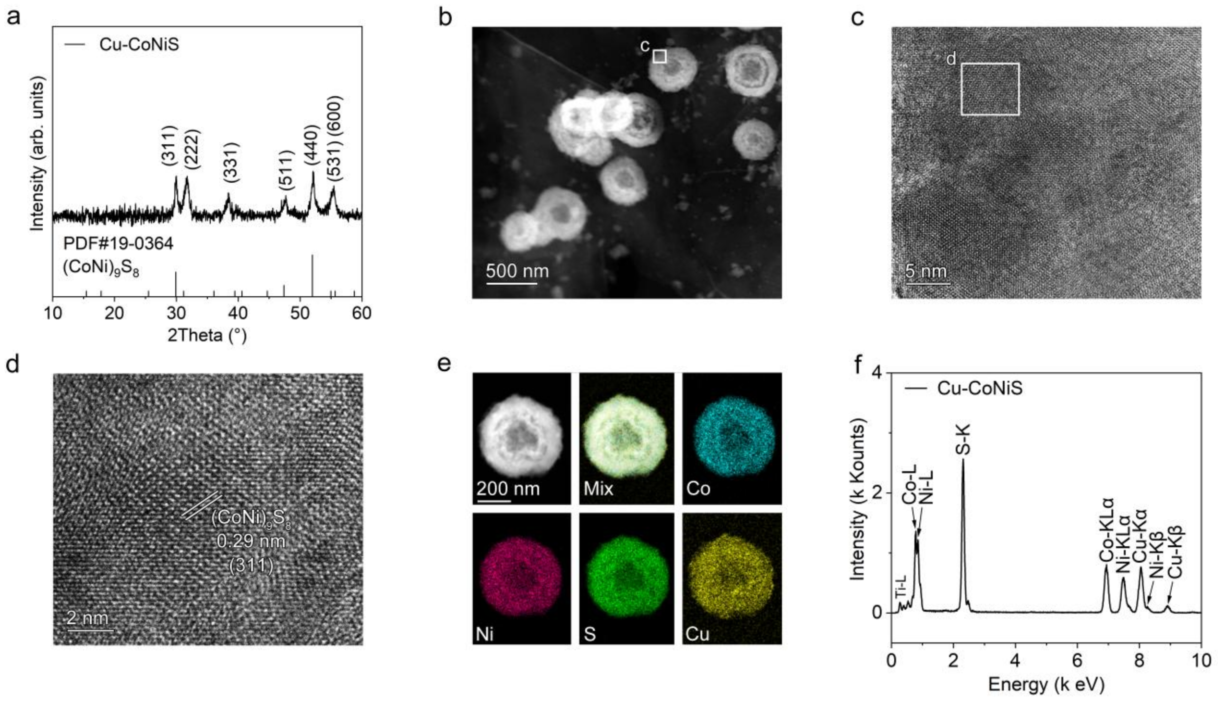

Supplementary Fig. 5 | Characterizations on Cu-CoNiS. a XRD pattern. **b** HAADF-STEM image. **c, d** HRTEM image. **e** HAADF-STEM image with elemental mapping. **f** EDS spectrum.

To explore the effects of metal doping, a Cu-doped CoNiS sample (Cu-CoNiS) was synthesized. XRD pattern confirms the main phase of $(CoNi)_9S_8$. Supplementary Fig. 5b shows that Cu doping induces a distinct bowl-like morphology. Multiple randomly oriented lattice fringes confirm its polycrystalline structure (Supplementary Fig. 5c). The HRTEM image shows lattice fringes with a spacing of 0.29 nm, which can be indexed to the (311) planes of $(CoNi)_9S_8$ with Cu doping. Elemental mapping and EDS analysis reveal uniform distribution of Co, Ni, and Cu with a molar ratio of 1: 0.8: 0.3. Unlike Sn doping, Cu can be readily incorporated, which significantly alters the nanostructure. Since the nanobowls are polycrystalline, it is difficult to clearly distinguish between crystalline and amorphous regions, unlike in single-crystalline structures where the interface can be easily identified.

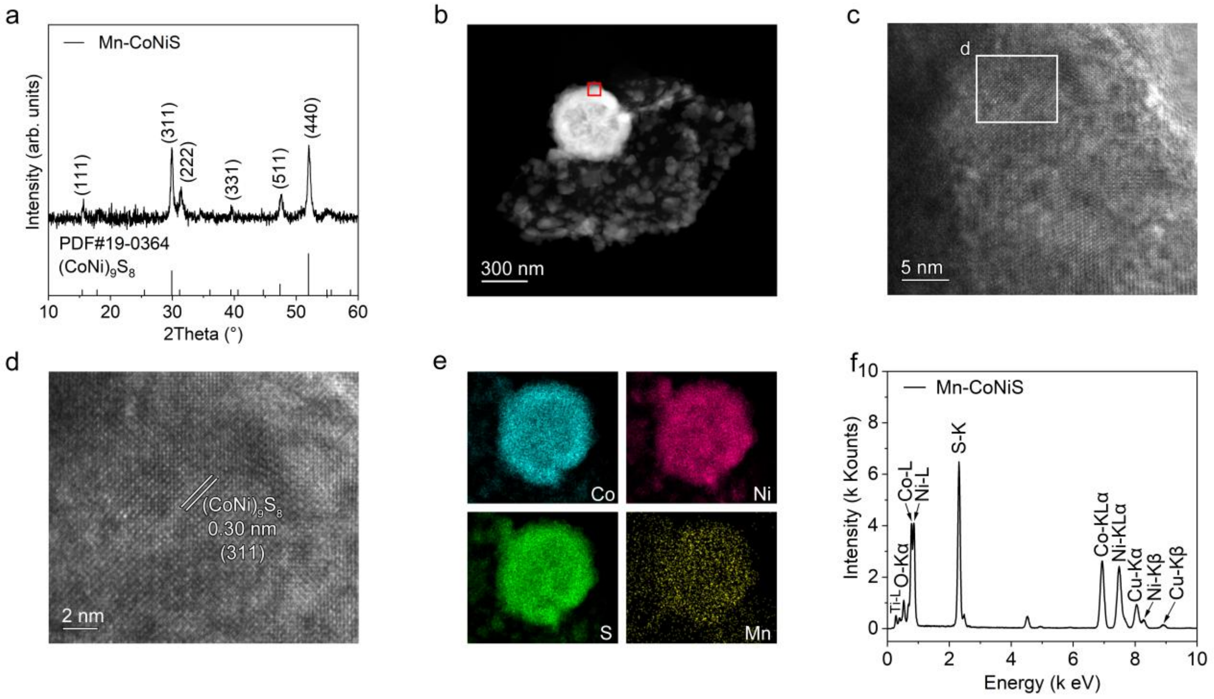

Supplementary Fig. 6 | Characterizations on Mn-CoNiS. a XRD pattern. **b** HAADF-STEM image. **c, d** HRTEM image. **e** Elemental mapping. **f** EDS spectrum.

To investigate the effect of metal doping, a Mn-doped CoNiS sample (Mn-CoNiS) was synthesized. XRD analysis (Supplementary Fig. 6a) confirms that the main phase remains $(CoNi)_9S_8$. The sample exhibits a bowl-like morphology, as shown in Supplementary Fig. 6b. Multiple randomly oriented lattice fringes confirm its polycrystalline structure (Supplementary Fig. 6c). HRTEM imaging (Supplementary Fig. 6d) reveals lattice fringes with a spacing of 0.30 nm, corresponding to the (311) plane of $(CoNi)_9S_8$ with Mn doping. Elemental mapping (Supplementary Fig. 6e) and EDS analysis (Supplementary Fig. 6f) reveal a uniform distribution of Co, Ni, and Mn with a molar ratio of 1: 1: 0.01. These results suggest that Mn was not effectively incorporated into the CoNiS lattice. The bowl is also polycrystalline, which is hard to distinguish the crystal and amorphous interfaces.

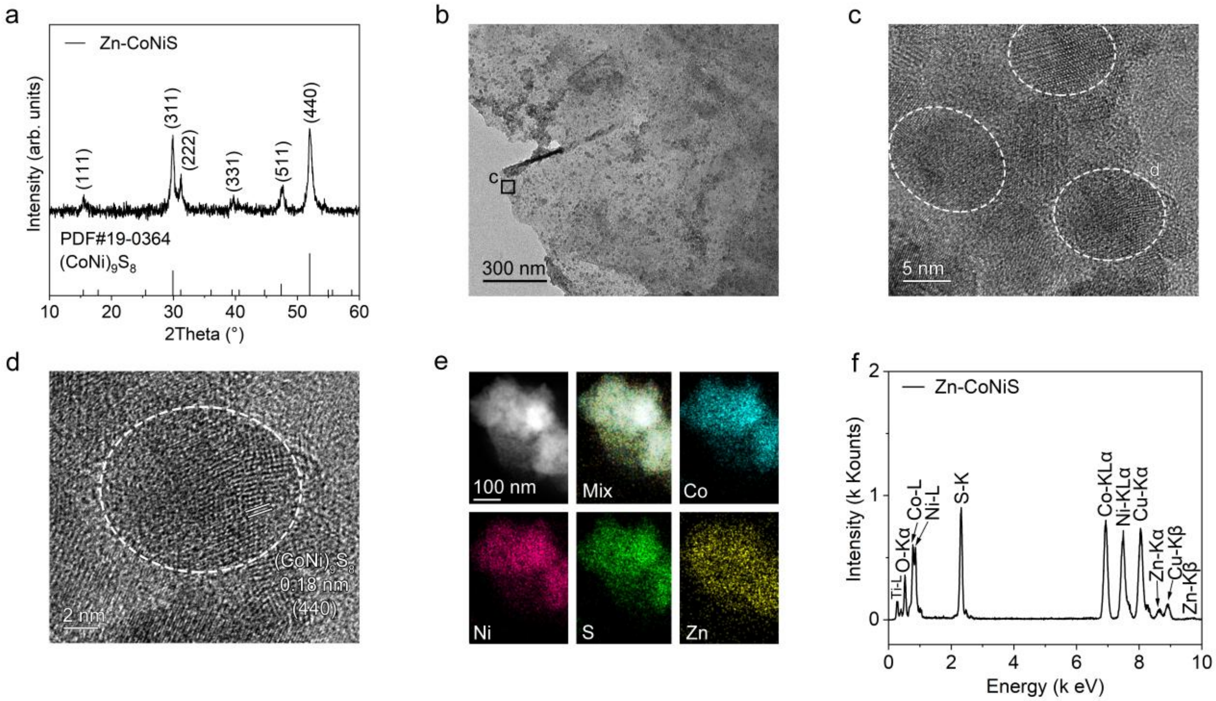

Supplementary Fig. 7 | Characterizations on Zn-CoNiS. a XRD pattern. **b** HAADF-STEM image. **c, d** HRTEM images. **e** HAADF-STEM image with elemental mapping. **f** EDS spectrum.

To explore the effects of metal doping, a Zn-doped CoNiS sample (Zn-CoNiS) was synthesized. XRD pattern confirms the main phase of $(CoNi)_9S_8$. Zn doping induces the formation of ~10 nm nanoparticles on the MXene surface (Supplementary Fig. 7b). The HRTEM image shows lattice fringes with a spacing of 0.18 nm, which can be indexed to the (440) planes of $(CoNi)_9S_8$. Elemental mapping and EDS analysis reveal uniform distribution of Co, Ni, and Zn with a molar ratio of 1: 0.9: 0.1. Unlike Sn doping, nanoparticles grown on MXene were synthesized by Zn doping.

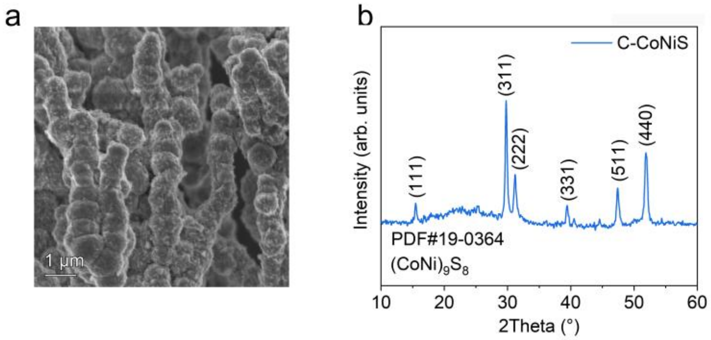

Supplementary Fig. 8 | Characterizations of C-CoNiS. a SEM image and **b** XRD pattern of C-CoNiS.

Supplementary Fig. 8a exhibits some linked spheres with an average diameter of 1 μm. In Supplementary Fig. 8b, the diffraction peaks match well with cubic Co_9S_8 structure with planes of (111), (311), (222), (331), (511), and (440) (JCPDS No. 19-0364). The sharp peaks show high degree of crystallinity.

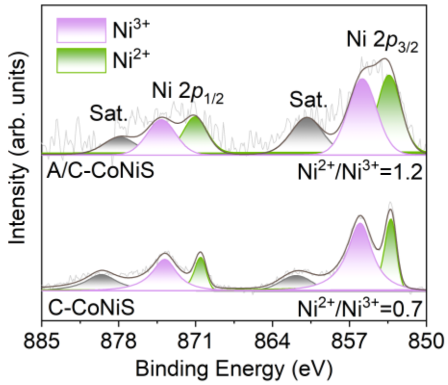

Supplementary Fig. 9 | High-resolution Ni 2*p* XPS spectra of A/C-CoNiS and C-CoNiS.

The Ni^{2+}/Ni^{3+} ratio increases from 0.7:1 (C-CoNiS) to 1.2:1 (A/C-CoNiS), demonstrating Sn doping modulates the electronic microenvironment of Ni atoms [4].

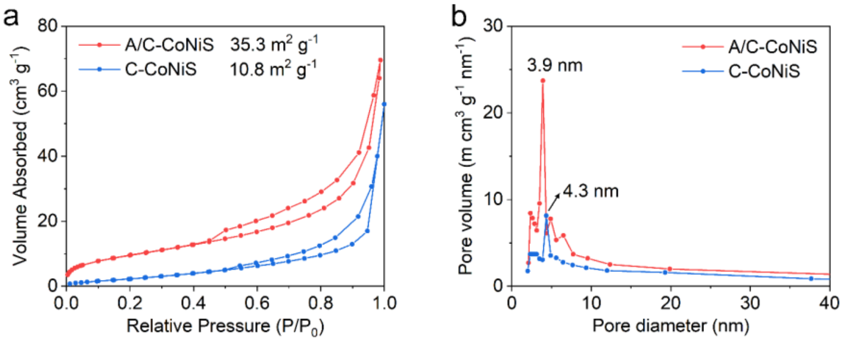

Supplementary Fig. 10 | Brunauer-emmett-teller (BET) characterization of A/C-CoNiS and C-CoNiS. a N_2 adsorption–desorption isotherms. **b** Pore size distribution calculated by the barrett-joyner-halenda (BJH) model.

Supplementary Fig. 10a shows that A/C-CoNiS exhibits a BET specific surface area of 35.3 $m^2\ g^{-1}$, much higher than that of C-CoNiS (10.8 $m^2\ g^{-1}$). The BJH pore size distribution in Supplementary Fig. 10b shows dominant mesopores centered at 3.9 nm for A/C-CoNiS and 4.3 nm for C-CoNiS, confirming a typical mesoporous structure. The two samples have similar pore size but A/C-CoNiS has more pores by comparing the pore volume. This mesoporous architecture facilitates electrolyte infiltration, accelerates ion transport, and provides abundant active sites, contributing to the improved electrochemical performance.

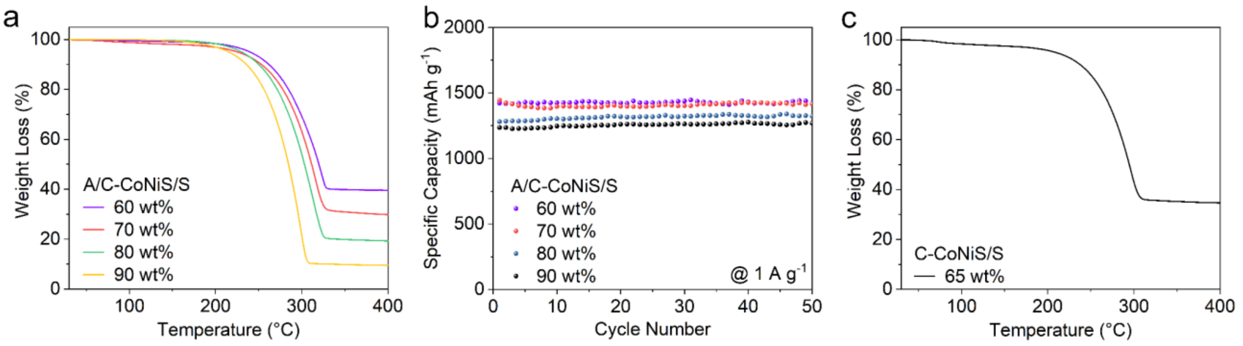

Supplementary Fig. 11 | a TGA profiles of A/C-CoNiS/S. **b** Cycling performance of A/C-CoNiS/S at sulfur loadings of 60 wt%, 70 wt%, 80 wt%, and 90 wt%. **c** TGA profile of C-CoNiS/S. TGA tests were conducted over the temperature range of 30 to 400 °C with a heating rate of 10 °C min^{-1}. No significant mass loss was observed before 55 °C. The rapid weight loss observed between 150 and 350 °C is attributed to the volatilization of sulfur. TGA measurements on A/C-CoNiS/S prepared at 9:1, 8:2, 7:3, and 6:4 mass ratios yield sulfur loadings of 90, 80, 70, and 60 wt%, respectively. Electrochemical cycling at 1 A g^{-1} (Supplementary Fig. 11b) shows that A/C-CoNiS/S with sulfur loadings of 60, 70, 80, and 90 wt% delivers capacities of 1419.9, 1417.0, 1323.6, and 1269.1 mAh g^{-1} after 50 cycles The results clearly show reveal no appreciable capacity difference at sulfur loadings of 60 % and 70 %. However, once the sulfur content reaches 80 % or 90 %, a discernible capacity fade is observed. Therefore, we selected a sulfur loading of 70 % for A/C-CoNiS/S. Furthermore, TGA measurement on C-CoNiS/S prepared at a 7:3 mass ratio show that the sulfur loading is only 65 wt% due to its limited adsorption capability (Supplementary Fig. 11c).

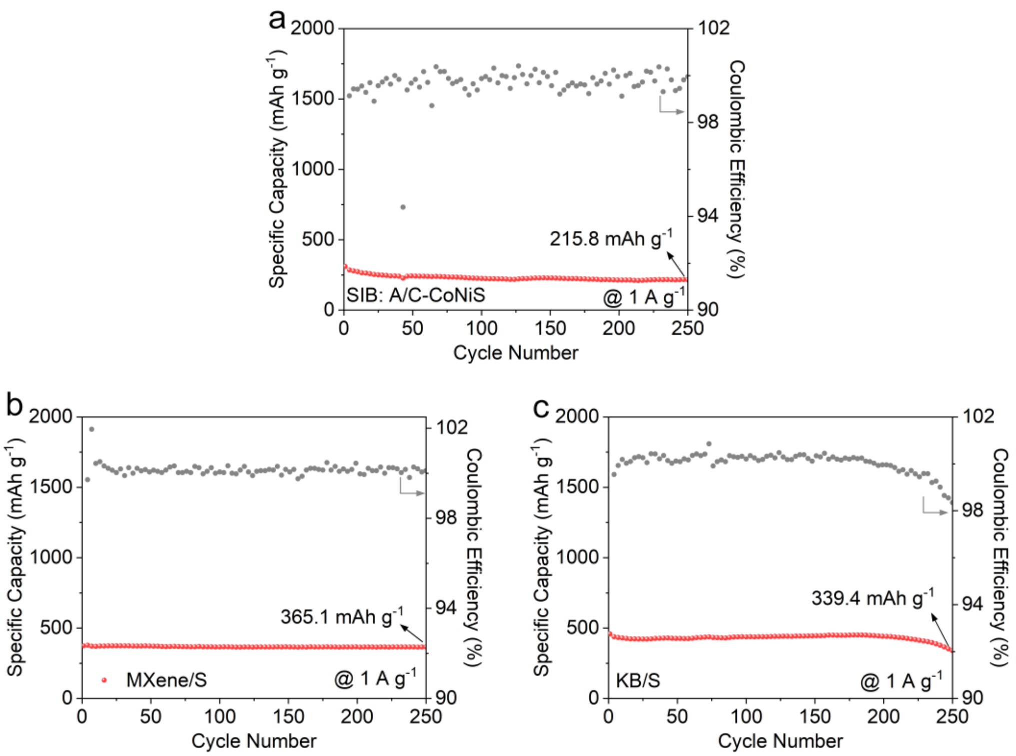

Supplementary Fig. 12 | Batteries performance using A/C-CoNiS, MXene/S, and KB/S at 1 A g^{-1}.

In sodium-ion batteries, A/C-CoNiS delivers 215.8 mAh g^{-1} after 250 cycles at 1 A g^{-1} (Supplementary Fig. 12a). Considering its 30 wt% content in the A/C-CoNiS/S composite, A/C-CoNiS contributes only 6.4 % of the total capacity, confirming that sulfur is the primary capacity source. In Na−S batteries, MXene/S and KB/S electrodes deliver discharge capacities of 365.1 and 339.4 mAh g^{-1} respectively after 250 cycles at 1 A g^{-1} (Supplementary Figs. 12b, c), highlighting the enhanced catalytic activity of A/C–CoNiS.

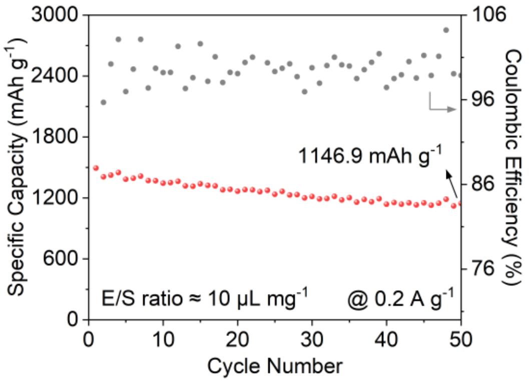

Supplementary Fig. 13 | Cycling performance of A/C-CoNiS/S at E/S ratio of 10 μL mg^{-1}.

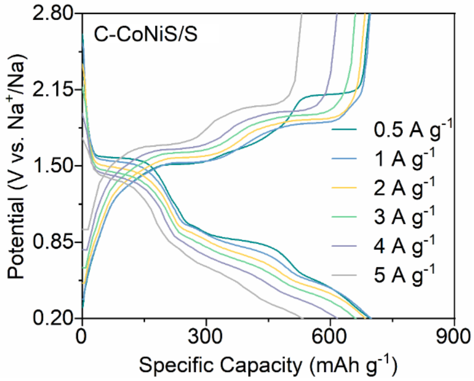

Supplementary Fig. 14 | Galvanostatic charge-discharge (GCD) curves of C-CoNiS/S at specific currents ranging from 0.5 to 5 A g^{-1}.

C-CoNiS/S exhibits rapid capacity decay and poorly defined voltage plateaus at higher currents as the specific current increases, revealing the C-CoNiS/S electrode has poor resistance to changes in current and slower reaction kinetics [5].

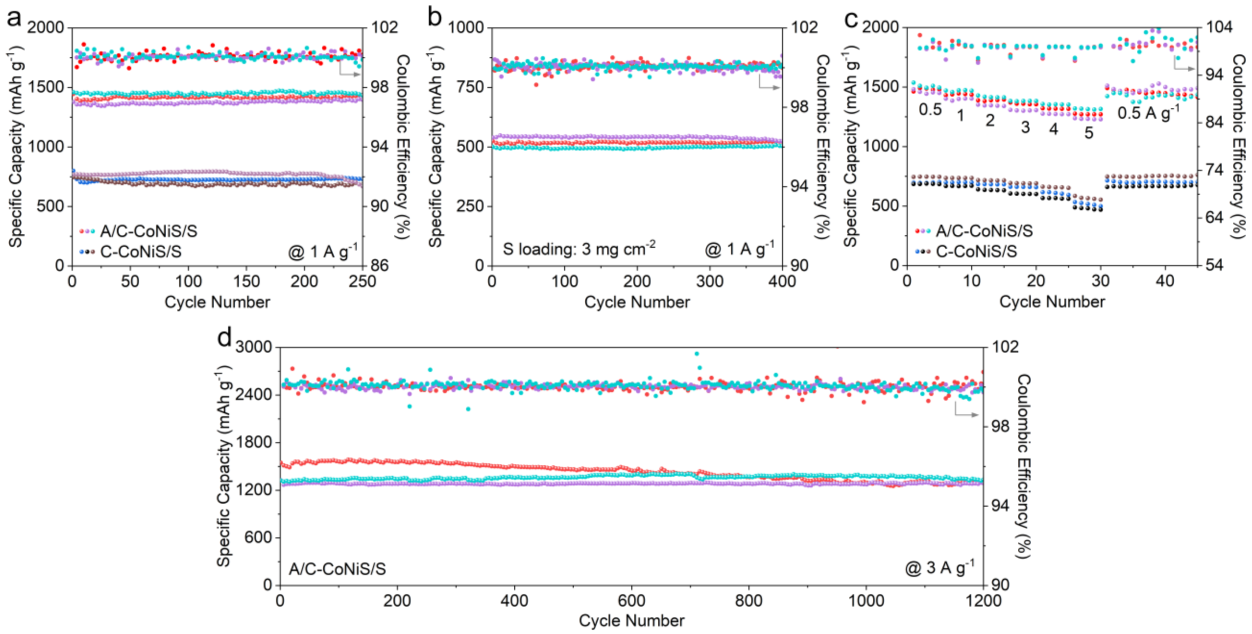

Supplementary Fig. 15 | Sodium-sulfur (Na||S) battery performance at room temperature. a Cycling performance at 1 A g^{-1}. **b** Cycle stability of 3mg cm^{-2} S loading. **c** Rate performance. **d** Long-term cycling stability. Data from three parallel cells are presented, confirming the reproducibility of the battery performance.

Supplementary Table 2.

Comparison of battery performance using A/C-CoNiS/S with recently reported room-temperature Li/Na||S batteries.

Electrode material	Specific current (A g^{-1})	Cycles (n)	Specific capacity after cycles (mAh g^{-1})	Retention (%)	Decay rate per cycle (%)	Reference
**#A/C-CoNiS/S**	**3**	**1200**	**1320.8**	**85.4**	**0.012**	**This work**
Co-PCL/S[6]	0.17	300	856	74.6	0.085	Nat. Commun. 2025
SA Co-N/S[7]	2	300	596	75.5	0.1	Nat. Commun. 2025
Co-GCN/S[8]	5	1000	405.1	47.5	0.053	Angew. Chem. Int. Ed. 2025
#S/NHC-InN_5 SAC[9]	1	800	384.9	60.0	0.050	Angew. Chem. Int. Ed. 2025
Bi_2Te_3/TiO_2-PP[10]	1.67	1000	735	77.9	0.028	Angew. Chem. Int. Ed. 2025
PSN-MXene[11]	3.4	800	450	60.8	0.049	Adv. Mater. 2025
#Zn−N_3O/HCs@S[12]	1.67	1000	1016	81.4	0.019	Adv. Funct. Mater. 2025
#Co-S-C@MC[13]	0.84	2500	910	77.5	0.009	Adv. Funct. Mater. 2025
#$FeSnO_x$@MXene[14]	0.84	400	610.3	63.2	0.092	Angew. Chem. Int. Ed. 2024
SP-Fe_3O_4-C[15]	1.67	1200	652.9	67.6	0.027	Nat. Commun. 2024
$BiVO_4$/S[16]	1.67	1000	910	83.0	0.017	Adv. Mater. 2024
P-$CoSe_2$/MXene[17]	1.67	500	625	66.5	0.067	Adv. Energy Mater. 2024
#Co-NMCN[18]	0.84	800	520	55.7	0.064	Adv. Funct. Mater. 2024
#S@MoS_2-Mo_1/SGF[19]	0.1	1000	505	50.0	0.050	Adv. Mater. 2023
#CoS_2/C[20]	0.84	800	678.9	89.4	0.013	Nat. Commun. 2020
#NiS_2/NPCTs[21]	1	750	401	41.8	0.078	Nat. Commun. 2019

Note: Na||S batteries are marked by hashtag (#), and the remains are Li||S batteries.

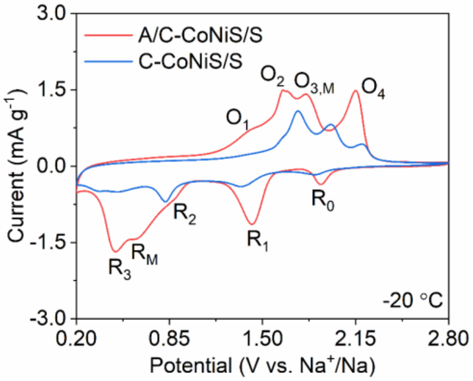

Supplementary Fig. 16 | CV curves of A/C-CoNiS/S and C-CoNiS/S at 0.2 mV s^{-1} at –20 °C.

Four prominent cathodic peaks are observed at 1.91 V (R_0), 1.43 V (R_1), 0.89 V (R_2), and 0.48 V (R_3), along with a distinct peak at 0.61 V (R_M). These peaks are sequentially associated with the stepwise reduction of sulfur species: R_0 corresponds to the initial conversion of S_8 into long-chain polysulfides (Na_2S_x, $6<x\leq8$); R_1 to the transition from long-chain Na_2S_x to Na_2S_4; R_2 and R_3 to the further reduction of Na_2S_4 to Na_2S. The peak R_M is attributed to Na^+ insertion into CoNiS [22]. During the anodic sweep, the peaks labeled O_1 to O_4 are assigned to the progressive reoxidation of Na_2S back to Na_2S_x. The peak O_M arises from the extraction of Na^+. Furthermore, A/C-CoNiS/S exhibits a larger current response and smaller polarization, indicating its favorable electrochemical behavior at –20 °C.

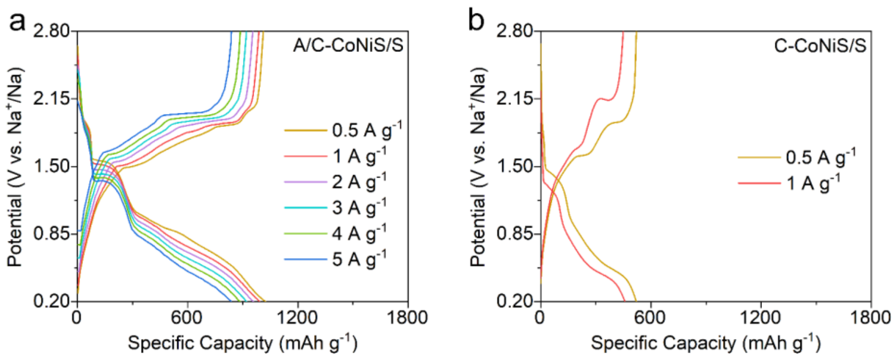

Supplementary Fig. 17 | GCD curves at −20 °C. a GCD curves of A/C-CoNiS/S with specific currents ranged from 0.5 to 5 A g^{-1}. **b** GCD curves of C-CoNiS/S at 0.5 and 1 A g^{-1}.

The GCD curves of A/C-CoNiS/S exhibits high capacities with well-defined charge/discharge plateaus even, whereas C-CoNiS/S shows rapid capacity decay and poorly defined voltage plateaus, showing improved low-temperature electrochemical performance of A/C-CoNiS [5].

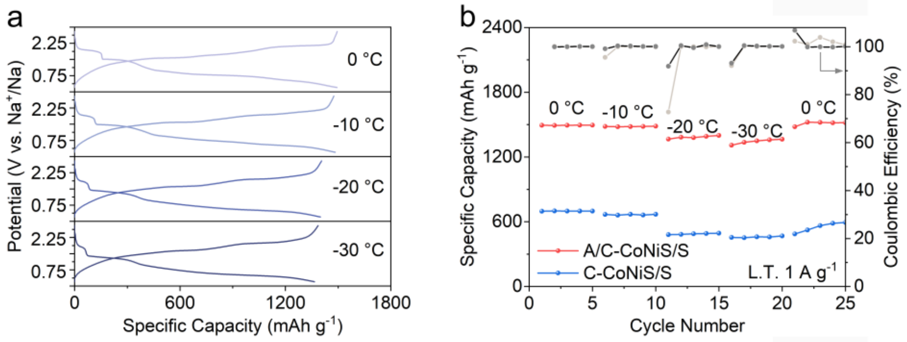

Supplementary Fig. 18 | Low-temperature electrochemical performance. a GCD curves of A/C-CoNiS/S from 0 °C to −30 °C at 1 A g^{-1}. **b** Cycling performance of A/C-CoNiS/S and C-CoNiS/S at low temperatures.

Supplementary Fig. 19a shows that the GCD curves of A/C-CoNiS/S exhibit distinct discharge plateaus at low temperatures. As the temperature decreases, the polarization voltage increases slightly. It reaching 0.89, 0.89, 0.95, and 1.01 V at 0 °C, −10 °C, −20 °C, and −30 °C, respectively. Supplementary Fig. 19b demonstrates that the specific capacities of A/C-CoNiS/S are 1493.4, 1483.3, 1399.2, and 1364.8 mAh g^{-1} at 0 °C, −10 °C, −20 °C, and −30 °C, respectively, highlighting its stable electrochemical behavior at low temperatures. By contrast, C-CoNiS/S exhibits significantly decreased capacities (697.8, 667.7, 479.6, and 454.8 mAh g^{-1}). A/C-CoNiS/S delivers specific capacities about 2~2.5 times the values of C-CoNiS/S at the same temperatures, clearly demonstrating the enhanced reaction kinetics of A/C-CoNiS at low temperatures.

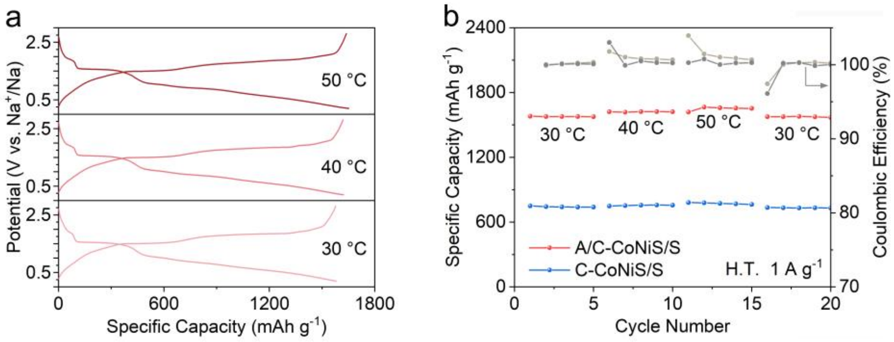

Supplementary Fig. 19 | Cycling performance of A/C-CoNiS/S and C-CoNiS/S at high temperatures.

The A/C-CoNiS/S electrode delivers discharge capacities of 1576.5, 1621.3, and 1652.7 mAh g^{-1} at 1 A g^{-1} at 30 °C, 40 °C, and 50 °C, respectively. By contrast, C-CoNiS/S shows capacities of 738.6, 757.1, and 763.3 mAh g^{-1} accordingly. A/C-CoNiS/S exhibits high capacities over 2 times of the values of its counterpart, demonstrating its stability and stable cycling behavior at high temperatures.

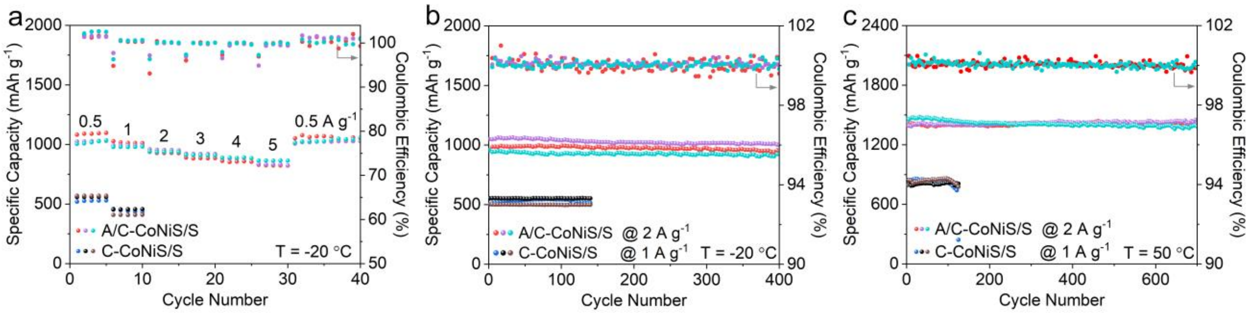

Supplementary Fig. 20 | Na||S battery performance at low and high temperatures. a Rate performance of A/C-CoNiS/S from 0.2 to 5 A g^{-1} at −20 °C. **b** Cycling performance for A/C-CoNiS/S and C-CoNiS/S at −20 °C. **c** Cycling performance of A/C-CoNiS/S and C-CoNiS/S at 50 °C.

Data from three parallel cells are presented, confirming the reproducibility of the battery performance.

Supplementary Table 3

Electrochemical performance of A/C-CoNiS/S compared to recently reported wide-temperature range Li/Na||S batteries.

Positive Electrode Material	Electrochemical performance at low temperature				Electrochemical performance at high temperature					Reference
	Temp. (°C)	Specific current (A g^{-1})	Cycle number (n)	Specific capacity after cycles (mAh g^{-1})	Temp. (°C)	Specific current (A g^{-1})	Cycle number (n)	Specific capacity after cycles (mAh g^{-1})	Decay rate per cycle (%)	
**#A/C-CoNiS/S**	**−20**	**2**	**400**	**949.9**	**50**	**2**	**700**	**1409.3**	**0.001**	**This work**
#Ni-B[23]	−10	2	1000	370	NA	–	–	–	–	Adv. Mater. 2024
$BiVO_4$/S[16]	−20	1.67	500	500	55	1.67	100	1038	0.26	Adv. Mater. 2024
Ni@C/CNT[24]	−40	0.17	100	280	60	3.34	500	534	0.086	Adv. Funct. Mater. 2023
MB-VN[25]	−10	1.67	100	553	60	3.35	200	668	0.17	ACS Nano 2023
SAF[26]	−40	0.17	100	313	60	0.87	100	672	0.39	Angew. Chem. Int. Ed. 2022
#ITO@ACC[27]	−10	0.17	100	342	NA	–	–	–	–	Energy Storage Mater. 2021

Note: Na||S batteries are marked by hashtag (#), and the remains are Li||S batteries.

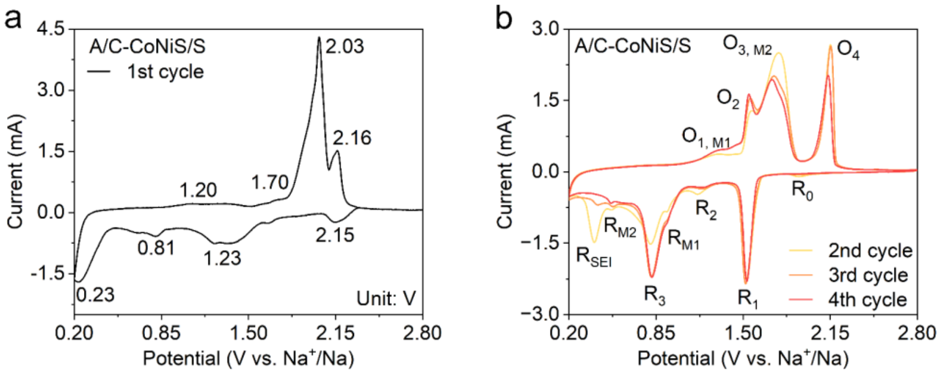

Supplementary Fig. 21 | CV curves of A/C-CoNiS/S at 0.2 mV s^{-1}.

During the initial cathodic scan, the reduction peaks at 2.15, 1.23, and 0.81 V correspond to the conversion of S_8 to long-chain polysulfides (Na_2S_x, $6<x\leq8$), the transformation of Na_2S_x to short-chain Na_2S_4, and the final reduction of Na_2S_4 to Na_2S, respectively [9,28-30]. In addition, the reduction peak at approximately 0.23 V is most likely attributable to the formation of the solid-electrolyte interphase (SEI) layer [31,32]. The oxidation peaks are associated with the stepwise oxidation of Na_2S to Na_2S_x [23]. In the CV curves of the later cycles, the reduction peaks related to sulfur species exhibit a noticeable shift and tend to stabilize, while the reduction peaks associated with the SEI gradually weaken and disappear by the fourth cycle [31,32]. Furthermore, the oxidation peaks split from a single peak into multiple peaks and eventually stabilize. The splitting of the oxidation peaks may be due to more thorough oxidation of Na_2S [33]. To investigate the properties of the battery during stable cycling, the CV data were collected from the fourth cycle unless otherwise specified.

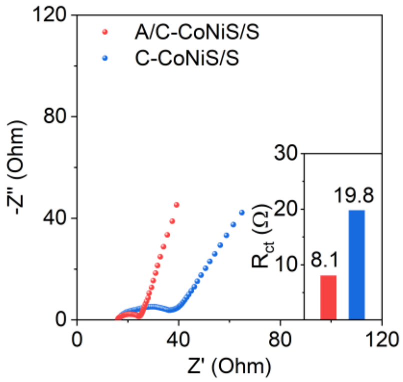

Supplementary Fig. 22 | Nyquist plots and corresponding R_{ct} values with the equivalent circuit model inserted.

The R_{ct} value of A/C-CoNiS/S is 8.1 Ω, much lower than that of C-CoNiS/S (19.8 Ω), indicating its faster charge transfer.

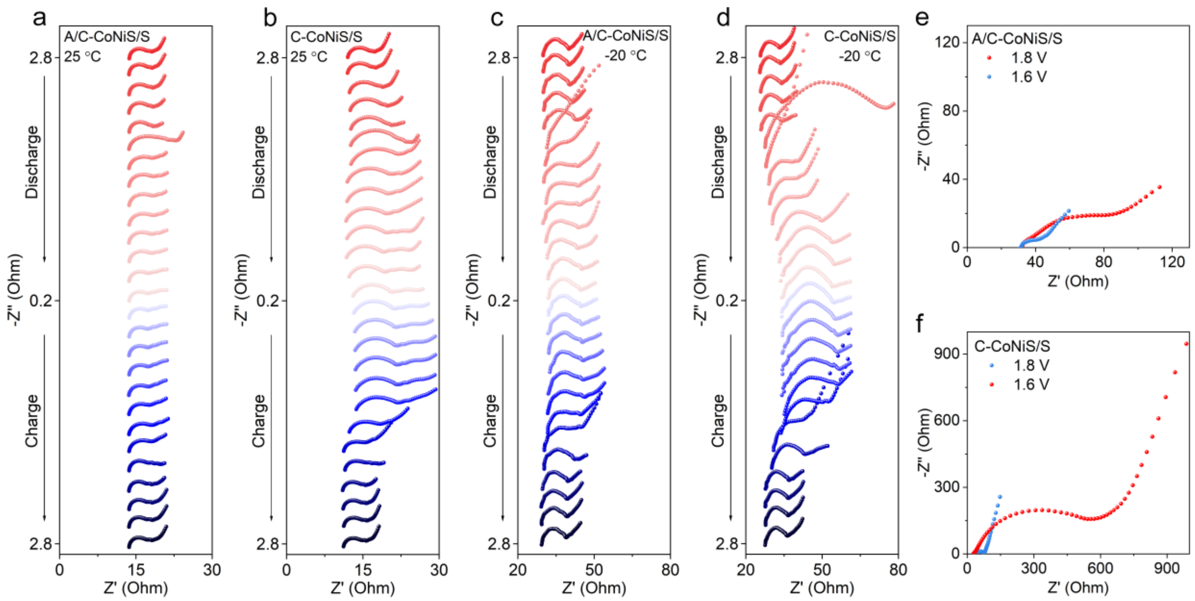

Supplementary Fig. 23 | In situ Electrochemical impedance spectroscopy (EIS) of Na||S batteries with different positive electrodes under various temperatures. a A/C-CoNiS/S at 25 °C, **b** C-CoNiS/S at 25 °C, **c** A/C-CoNiS/S at –20 °C, and **d** C-CoNiS/S at –20 °C. **e, f** Full-range impedance spectra at 1.6 and 1.8 V during discharge, corresponding to **c** and **d**.

In situ EIS was employed to monitor the reaction kinetics during discharge/charge process. The R_{ct} of A/C-CoNiS/S remains consistently lower than that of C-CoNiS/S at 25 °C by observing semicircle diameter. The difference between the two samples becomes more pronounced at −20 °C. Furthermore, the maximum value of R_{ct} for both A/C-CoNiS/S and C-CoNiS/S can be detected at ~1.8 V at 25 °C. However, at −20 °C, the peak R_{ct} of A/C-CoNiS/S remains at 1.8 V with a slight increase, while that of C-CoNiS/S shifts to 1.6 V and rises sharply, suggesting enhanced polarization and slower kinetics [34,35]. The results indicate A/C-CoNiS/S maintains stable charge transfer kinetics even at low temperature, demonstrating a favorable low-temperature adaptability [35].

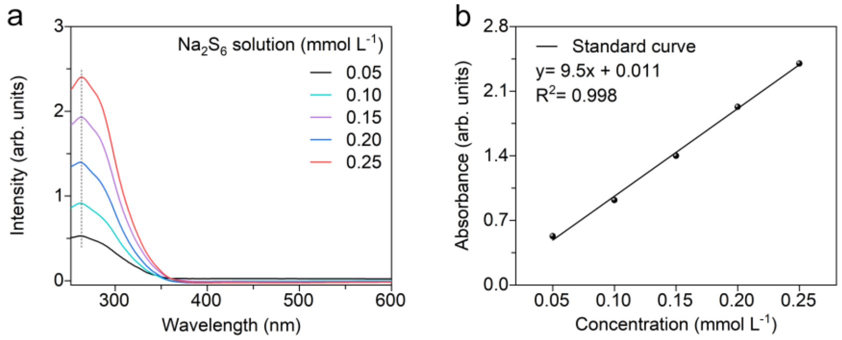

Supplementary Fig. 24 | Adsorption spectra of Na_2S_6 solutions with various concentrations and the resulting standard plot. a UV–vis absorption spectra of Na_2S_6 solutions with varying concentrations. **b** Corresponding calibration curve used for quantitative analysis.

Based on the established calibration curve, the adsorption capacity of the catalyst for polysulfides can be quantitatively determined from the corresponding UV–vis absorption data.

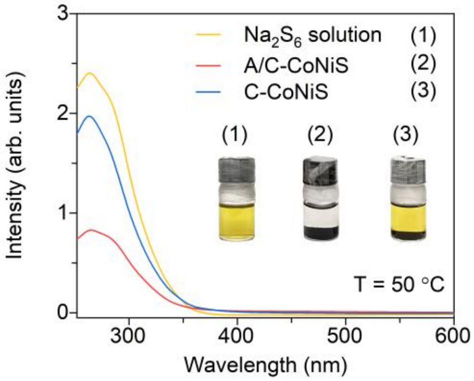

Supplementary Fig. 25 | UV-vis spectra of Na_2S_6 solutions after immersion with A/C-CoNiS and C-CoNiS at 50 °C for 12h. Insets: digital photographs of the solutions before and after adsorption. Compared to C-CoNiS and the Na_2S_6 solution, A/C-CoNiS exhibits weaker UV-vis signals and a more transparent solution (inset), indicating stronger sodium polysulfides (NaPSs) adsorption and improved anti-shuttle capability [4].

Supplementary Table 4.

Polysulfide adsorption capacity of A/C-CoNiS and C-CoNiS at different temperatures.

Sample	Temperature (ºC)	Adsorption Capacity ($mmol\cdot g^{-1}$)	Adsorption Capacity ($g_s\cdot g^{-1}$)
A/C-CoNiS	25	0.336	0.080
	50	0.328	0.078
C-CoNiS	25	0.235	0.056
	50	0.086	0.020

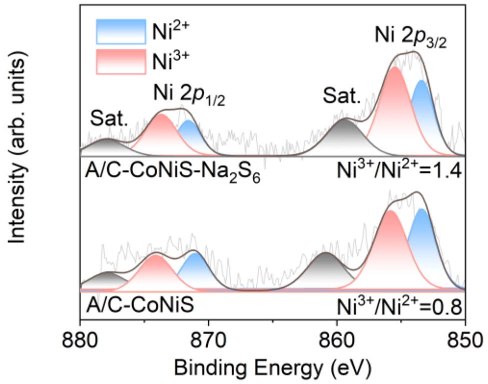

Supplementary Fig. 26 | High-resolution Ni 2*p* XPS spectra of A/C-CoNiS before and after adsorbing Na_2S_6.

The molar ratio of Ni^{3+}/Ni^{2+} increases from 0.8 to 1.4 after adsorbing Na_2S_6. This increase in the oxidation state of Ni indicates more Ni–S bonds formation with linking between Ni atoms and S atoms from NaPSs, suggesting that Ni atoms serve as the adsorption sites for NaPSs.

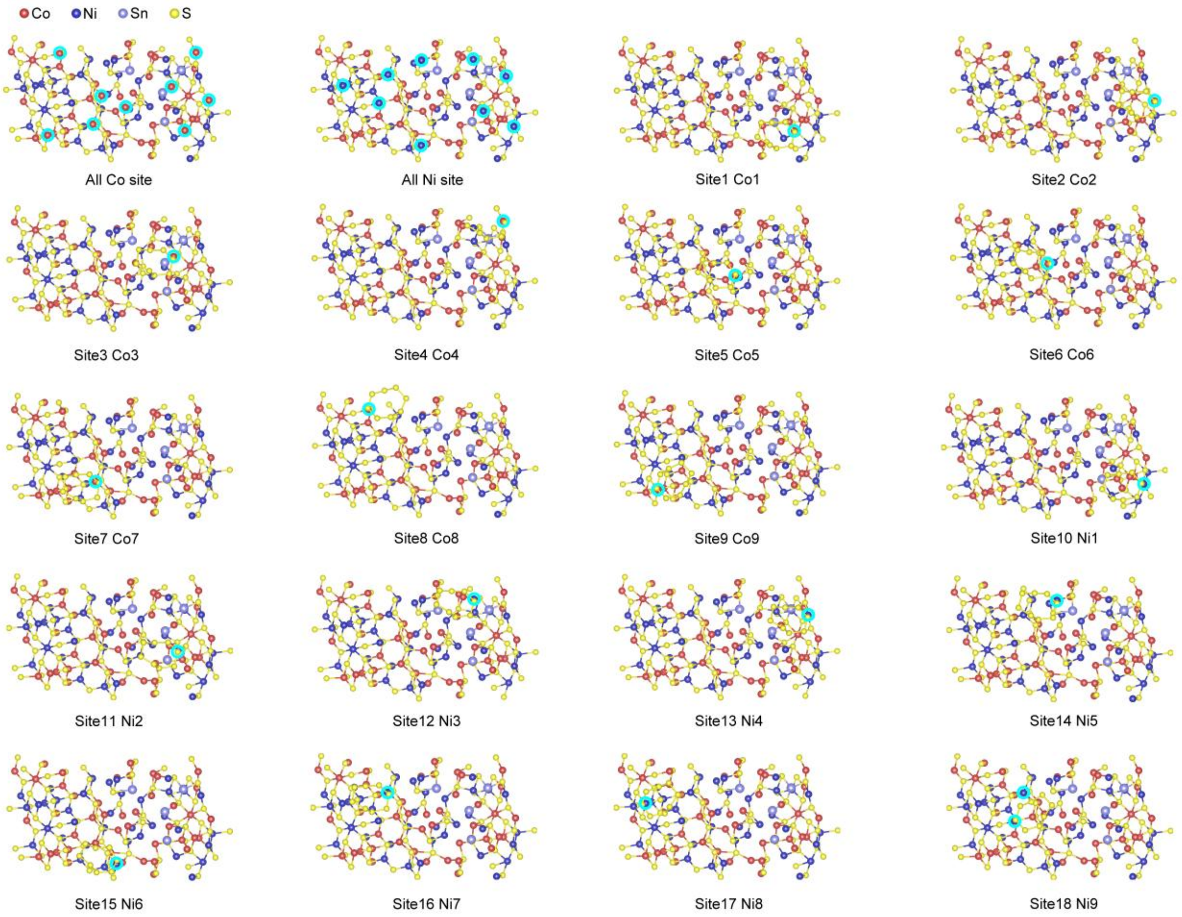

Supplementary Fig. 27 | Calculated adsorption free energy of S_8 on A/C-CoNiS at different adsorption sites.

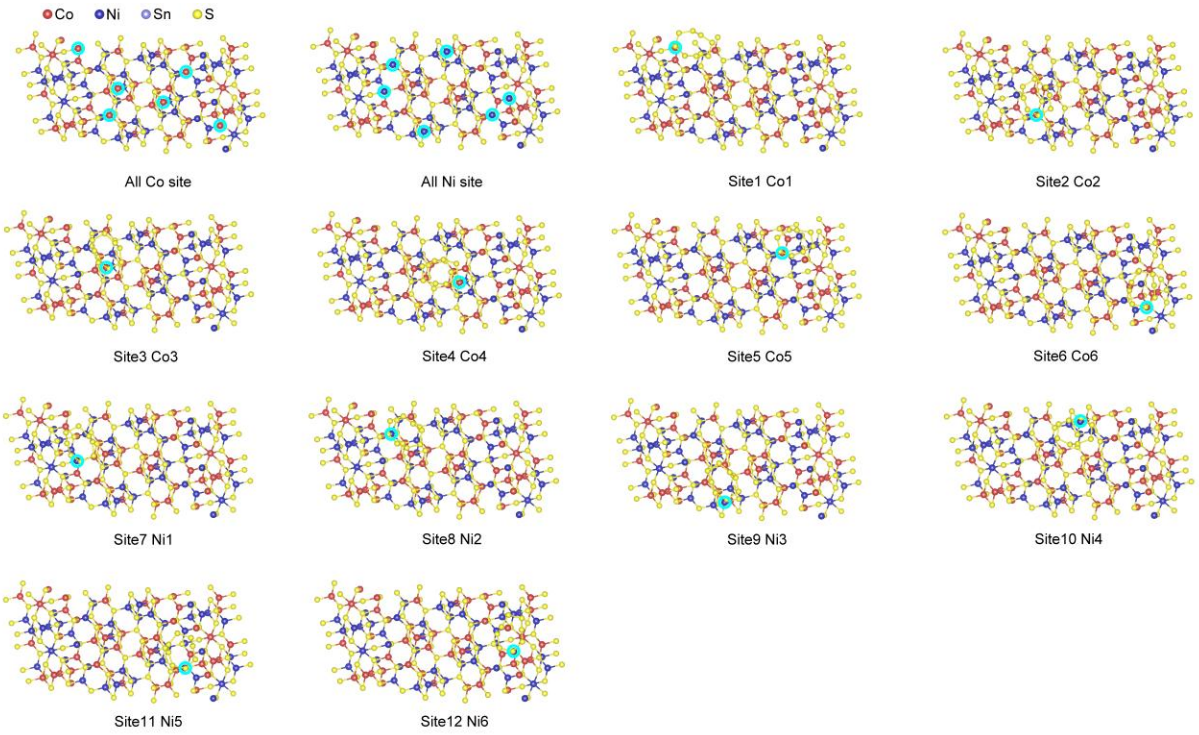

Supplementary Fig. 28 | Calculated adsorption free energy of S_8 on C-CoNiS at different adsorption sites.

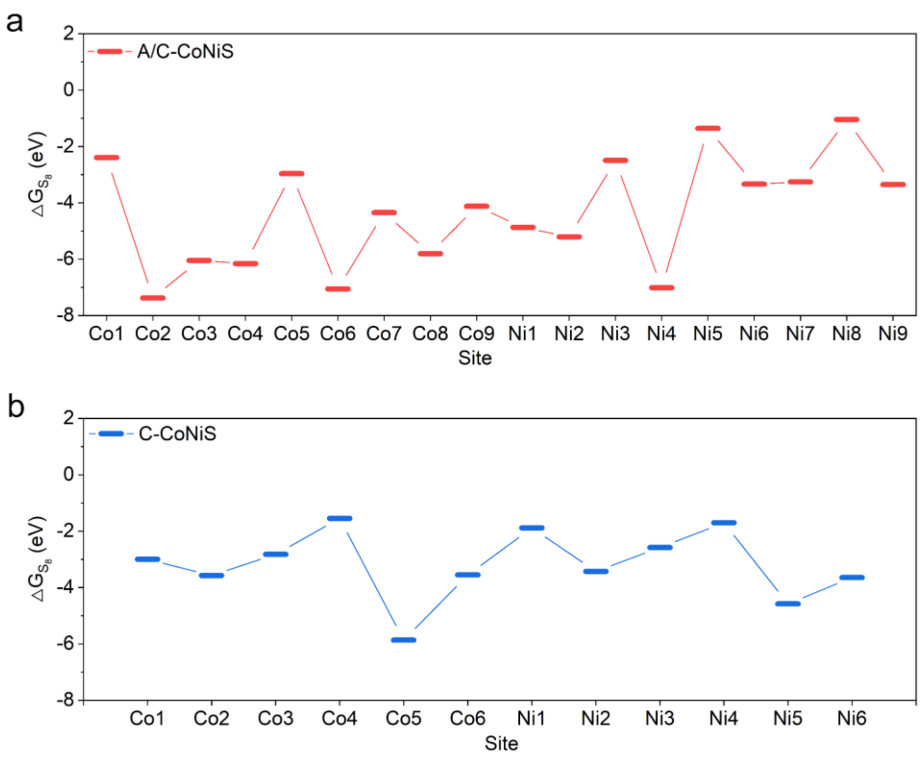

Supplementary Fig. 29 | Gibbs free energy profiles of S_8 adsorption at different sites on **a** C–CoNiS and **b** A/C–CoNiS.

Site Screening Methodology: The S_8 molecule was found to preferentially adsorb on transition-metal (Co, Ni) sites rather than sulfur sites. Accordingly, all exposed transition-metal atoms were evaluated, and the site with the strongest binding energy was identified as the active site for further investigation. The corresponding adsorption configurations and energies were provided in Supplementary Figs. 27–29.

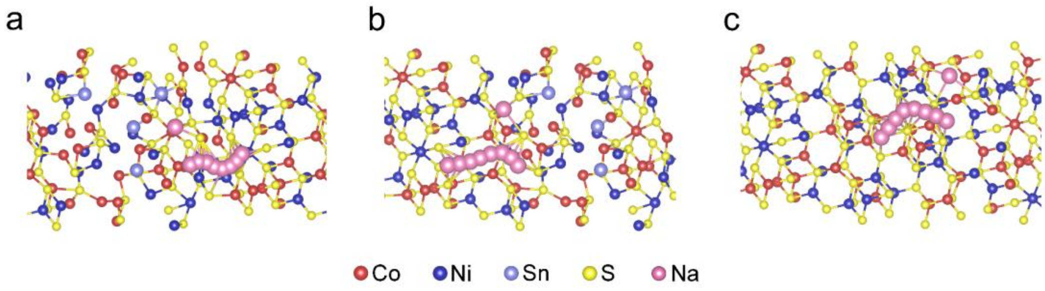

Supplementary Fig. 30 | CI-NEB calculation of the dissociation pathway of Na_2S on **a** Amor-A/C-CoNiS, **b** Cryst-A/C-CoNiS, and **c** C-CoNiS surfaces.

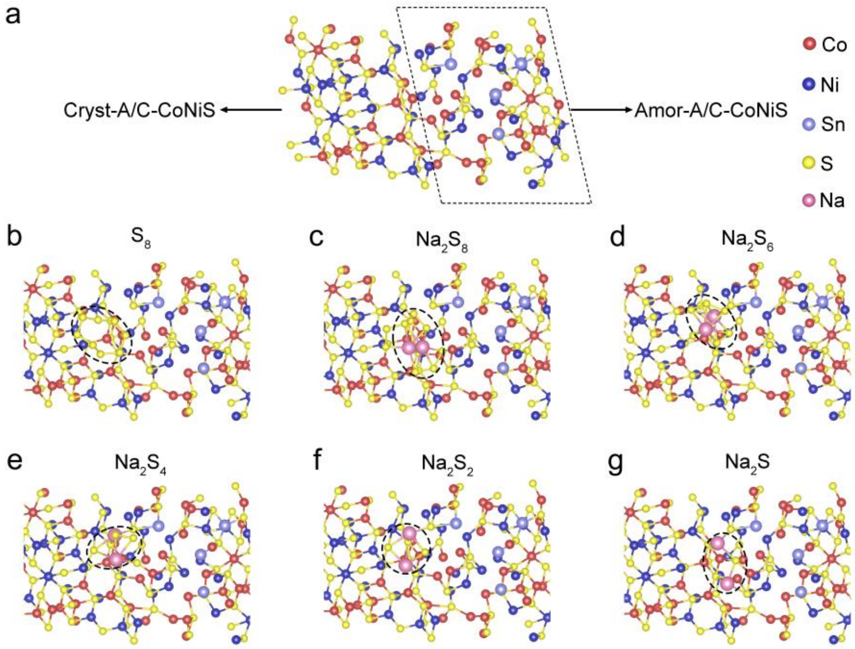

Supplementary Fig. 31 | Density functional theory (DFT)-optimized Cryst-A/C-CoNiS before and after adsorption of S_8 and NaPSs species. a Structural model of A/C-CoNiS. Adsorption configurations of **b** S_8, **c** Na_2S_8, **d** Na_2S_6, **e** Na_2S_4, **f** Na_2S_2, and **g** Na_2S molecules on the Cryst-A/C-CoNiS surface. Dashed circles highlight S_8 and NaPSs species.

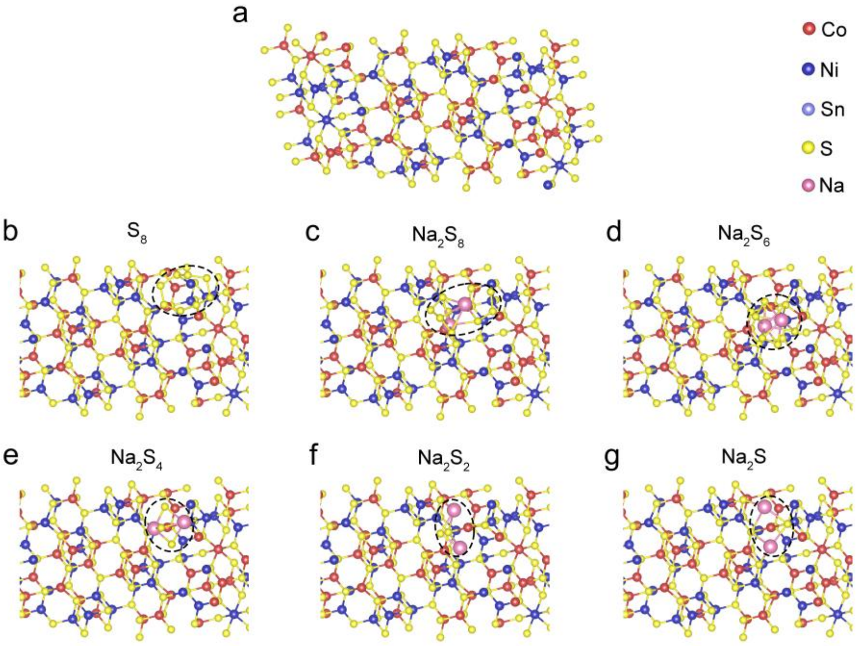

Supplementary Fig. 32 | C-CoNiS crystal structures before and after adsorbing S_8 and NaPSs species through DFT optimization. **a** Crystal structure model of C-CoNiS. Adsorption configurations of **b** S_8, **c** Na_2S_8, **d** Na_2S_6, **e** Na_2S_4, **f** Na_2S_2, and **g** Na_2S molecules on the C-CoNiS surface. Dashed circles highlight S_8 and NaPSs species.

Supplementary Table 5. Activation data for all electrochemical cycling tests.

Fig.	Positive Electrode Material	Specific current (A g^{-1})	Cycle number (n)	Specific capacity (mAh g^{-1})	Coulombic efficiency
2b	A/C-CoNiS/S	0.5	1	1568.8	
			2	1503.1	99.8
			3	1521.2	100.1
	C-CoNiS/S		1	738.4	
			2	752.1	99.6
			3	674.4	91.1
2c	A/C-CoNiS/S	0.5	1	1149.6	
			2	1067.2	99.5
			3	1087.5	99.9
2e	A/C-CoNiS/S	0.2	1	1624.8	
			2	1573.1	100.2
	C-CoNiS/S		1	671.1	
			2	796.2	96.9
2g	A/C-CoNiS/S	0.5	1	1632.5	
			2	1594.5	99. 2
			3	1566.1	97.9
3b	A/C-CoNiS/S	0.2	1	1137.7	
			2	1097	94.4
	C-CoNiS/S		1	213.8	
			2	462.1	88.2
3c	A/C-CoNiS/S	0.5	1	903.2	
			2	842.6	92.2
			3	973.2	107.6
	C-CoNiS/S		1	596.1	
			2	603.4	97.7
			3	571.2	103.3
3e	A/C-CoNiS/S	0.5	1	1605.2	
			2	1534.7	37.3
	C-CoNiS/S		1	904.1	
			2	861.3	180.2

References

1 Tao, X. et al. Ti_3C_2 QDs@CNTs with active titanium species as bidirectional catalytic cathode for facilitating lithium polysulfide conversion in Li–S batteries. *Adv. Funct. Mater.* **35**, 2420532 (2025).

2 Lu, C. et al. S-decorated porous Ti_3C_2 MXene combined with In situ forming Cu_2Se as effective shuttling interrupter in Na–Se batteries. *Adv. Mater.* **33**, e2008414 (2021).

3 Xiaodan Huang et al. Multimodal probing of T-cell recognition with hexapod heterostructures. *Nat. Methods* **21**, 857–867 (2024).

4 Jing, Z. et al. Rational design of prussian blue analogues for ultralong and wide-temperature-range sodium–ion batteries. *J. Am. Chem. Soc.* **147**, 3702−3713 (2025).

5 He, J., Bhargav, A., Su, L., Charalambous, H., Manthiram, A. Intercalation-type catalyst for non-aqueous room temperature sodium–sulfur batteries. *Nat. Commun.* **14**, 6568 (2023).

6 Kim, S. et al. Protective catalytic layer powering activity and stability of electrocatalyst for high-energy lithium–sulfur pouch cell. *Nat. Commun.* **16** (2025).

7 Bai, R. et al. Preferable single-atom catalysts enabled by natural language processing for high energy density Na-S batteries. *Nat. Commun.* **16**, 5827 (2025).

8 You, T. Q. et al. Insights into Co-catalytic single-atom-support interactions for boosting sulfur reduction electrocatalysis. *Angew. Chem. Int. Ed.*, e202425144 (2025).

9 Wu, G. X. et al. Optimizing s–p orbital overlap between sodium polysulfides and single-atom indium catalyst for efficient sulfur redox reaction. *Angew. Chem. Int. Ed.*, e202422208 (2025).

10 Chen, D. H. et al. Topological insulator heterojunction with electric dipole domain to boost polysulfide conversion in lithium-sulfur batteries. *Angew. Chem. Int. Ed.*, e202423357 (2025).

11 Luo, Z. H. et al. 2D nanochannel interlayer realizing high-performance lithium-sulfur batteries. *Adv. Mater.*, 2417321 (2025).

12 Zheng, F. C. et al. Template-sacrificing synthesis of asymmetrically coordinated Zn single-

atom sites for high-performance sodium–sulfur batteries. *Adv. Funct. Mater.* **35**, 2413084 (2025).

13 Wei, X. L., Zhang, Z., Luo, D., Wang, X. Construction of heterointerfaced nanoreactor electrocatalyst via in situ evolution toward practical room-temperature sodium–sulfur batteries. *Adv. Funct. Mater.* **35**, 2414172 (2025).

14 Sun, W. et al. Amorphous $FeSnO_x$ nanosheets with hierarchical vacancies for room-temperature sodium–sulfur batteries. *Angew. Chem. Int. Ed.* **63**, e202404816 (2024).

15 Zhang, H. et al. Fe_3O_4-doped mesoporous carbon cathode with a plumber's nightmare structure for high-performance Li–S batteries. *Nat. Commun.* **15**, 5451 (2024).

16 Deng, D. R. et al. Accelerating the rate-determining steps of sulfur conversion reaction for lithium–sulfur batteries working at an ultrawide temperature range. *Adv. Mater.* **36**, 2406135 (2024).

17 Wang, J. et al. Lattice strain and charge localization dual regulation of phosphorus-doped $CoSe_2$/MXene catalysts enable kinetics-enhanced and dendrite-free lithium–sulfur batteries. *Adv. Energy Mater.* **14**, 2401630 (2024).

18 Mei, T. H. et al. Cobalt catalytic regulation engineering in room-temperature sodium–sulfur batteries: facilitating rapid polysulfides conversion and delicate Na_2S nucleation. *Adv. Funct. Mater.* **35**, 2418126 (2024).

19 Zhang, B. W. et al. Atomically dispersed dual-site cathode with a record high sulfur mass loading for high-performance room-temperature sodium–sulfur batteries. *Adv. Mater.* **35**, 2206828 (2023).

20 Aslam, M. K. et al. Metal chalcogenide hollow polar bipyramid prisms as efficient sulfur hosts for Na–S batteries. *Nat. Commun.* **11**, 5242 (2020).

21 Yan, Z. et al. Nickel sulfide nanocrystals on nitrogen-doped porous carbon nanotubes with high-efficiency electrocatalysis for room-temperature sodium–sulfur batteries. *Nat. Commun.* **10**, 4793 (2019).

22 Li, Q. et al. Optimized Co-S bonds energy and confinement effect of hollow MXene@CoS/NC for enhanced sodium storage kinetics and stability. *Chem. Eng. J.* **450**,

137922 (2022).

23 Wang, B. et al. In situ electrochemical evolution of amorphous metallic borides enabling long cycling room-/subzero-temperature sodium–sulfur batteries. *Adv. Mater.* **36**, e2411725 (2024).

24 Zhang, H. et al. Operating lithium–sulfur batteries in an ultrawide temperature range from –50 °C to 70 °C. *Adv. Funct. Mater.* **33**, 2304433 (2023).

25 Ma, L. et al. Wide-temperature operation of lithium–sulfur batteries enabled by multi-branched vanadium nitride electrocatalyst. *ACS Nano* **17**, 11527–11536 (2023).

26 Xu, J. et al. Realizing all-climate Li–S batteries by using a porous sub-nano aromatic framework. *Angew. Chem. Int. Ed.* **61**, e202211933 (2022).

27 Kumar, A. et al. Sub-zero and room-temperature sodium–sulfur battery cell operations: a rational current collector, catalyst and sulphur-host design and study. *Energy Storage Mater.* **42**, 608–617 (2021).

28 Lei, Y. et al. Streamline sulfur redox reactions to achieve efficient room-temperature sodium-sulfur batteries. *Angew. Chem. Int. Ed.* **61**, e202200384 (2022).

29 Li, C. et al. Three birds with one stone: multifunctional separators based on SnSe nanosheets enable high-performance Li–, Na– and k–sulfur batteries. *Adv. Energy Mater.* **14**, 2303551 (2024).

30 Zou, Y. et al. Nitrogen and Sulfur Vacancies in Carbon Shell to Tune Charge Distribution of $Co_6Ni_3S_8$ Core and Boost Sodium Storage. *Adv. Energy Mater.* **10**, 1904147 (2020).

31 Cheng, D. et al. Engineering ultrathin carbon layer on porous hard carbon boosts sodium storage with high Initial coulombic efficiency. *ACS Nano* **17**, 19063-19075 (2023).

32 Yan, Z. et al. A high-kinetics sulfur cathode with a highly efficient mechanism for superior room-temperature Na–S batteries. *Adv. Mater.* **32**, e1906700 (2020).

33 Li, C. et al. Deep Eutectic Solvent Binder Facilitating Reaction Kinetics of Lithium Sulfur Batteries. *Angew. Chem. Int. Ed.* **64**, e202516009 (2025).

34 Xu, M. Y. et al. Atom-dominated relay catalysis of high-entropy MXene promotes cascade

polysulfide conversion for lithium-sulfur batteries. *Energy Environ. Sci.* **17**, 7735-7748 (2024).

35 Li, C. H. et al. Balancing Electronic spin state via atomically-dispersed heteronuclear Fe-Co pairs for high-performance sodium-sulfur batteries. *J. Am. Chem. Soc.* **147**, 8250-8259 (2025).
