# Supplementary material for: Amorphous/crystalline interwoven multipods with high Co/Ni activity for wide-temperature-range sodium-sulfur batteries
Source: Nat Commun. 2026 Mar 10;17:2333. doi: 10.1038/s41467-026-69749-7 (PMC12979862; doi:10.1038/s41467-026-69749-7)
Supplement: Supplementary file 2 — Description of Additional Supplementary Files [file 41467_2026_69749_MOESM2_ESM.pdf]

Description of Additional Supplementary Files

Supplementary Data:

1. Pristine material structures: Files 1–2 record optimized structure models of the pristine A-AC-CONIS (File 1) and C-CONIS (File 2) materials.

2. Surface-supported systems: Files 3–20 present optimized structure models of Na_2S/Na_2S_2/Na_2S_4/Na_2S_6/Na_2S_8/S_8 adsorbed on A-AC-CONIS, C-AC-CONIS, or C-CONIS surfaces (specific combinations align with file numbering).

3. NEB process structure models: Files 21–23 contain the complete set of optimized structure models corresponding to the entire NEB calculation process (including initial, final, and intermediate images) for the A-AC-CONIS (File 21), C-AC-CONIS (File 22), and C-CONIS (File 23) systems, respectively.
